# The effects of postnatal maternal depression and anxiety on the processing of infant faces

**DOI:** 10.1016/j.jad.2011.04.015

**Published:** 2011-09

**Authors:** Adriane Arteche, Jutta Joormann, Allison Harvey, Michelle Craske, Ian H. Gotlib, Annukka Lehtonen, Nicholas Counsell, Alan Stein

**Affiliations:** a Department of Psychiatry, University of Oxford, UK; b Department of Psychology, University of Miami, USA; c Department of Psychology, University of California, Berkeley, USA; d Department of Psychology, University of California, Los Angeles, USA; e Department of Psychology, Stanford University, USA

**Keywords:** Depression, Infant faces, Morphed faces, Mother–child interaction

## Abstract

**Background:**

Postnatally depressed mothers have difficulties responding appropriately to their infants. The quality of the mother–child relationship depends on a mother's ability to respond to her infant's cues, which are largely non-verbal. Therefore, it is likely that difficulties in a mother's appraisal of her infants' facial expressions will affect the quality of mother–infant interaction. This study aimed to investigate the effects of postnatal depression and anxiety on the processing of infants' facial expressions.

**Method:**

A total of 89 mothers, 34 with Generalised Anxiety Disorder, 21 with Major Depressive Disorder, and 34 controls, completed a ‘morphed infants’ faces task when their children were between 10 and 18 months.

**Results:**

Overall, mothers were more likely to identify happy faces accurately and at lower intensity than sad faces. Depressed compared to control participants, however, were less likely to accurately identify happy infant faces. Interestingly, mothers with GAD tended to identify happy faces at a lower intensity than controls. There were no differences between the groups in relation to sad faces.

**Limitations:**

Our sample was relatively small and further research is needed to investigate the links between mothers' perceptions of infant expressions and both maternal responsiveness and later measures of child development.

**Conclusion:**

Our findings have potential clinical implications as the difficulties in the processing of positive facial expressions in depression may lead to less maternal responsiveness to positive affect in the offspring and may diminish the quality of the mother–child interactions. Results for participants with GAD are consistent with the literature demonstrating that persons with GAD are intolerant of uncertainty and seek reassurance due to their worries.

## 1 Introduction

There is growing evidence that postnatally depressed mothers have difficulties responding appropriately to their infants (see Murray et al., 2010, for a review of this literature). Although these difficulties may adversely influence subsequent child development (Yarrow et al., 1984), including problems in infant learning, attention, language, and emotional regulation (Goodman, 2004; Murray et al., 2010; Stein et al., 2008), the mechanisms involved in this process have not been fully elucidated. In particular, the question of *whether* and *how* depression-associated cognitive biases may explain the adverse effects of postnatal depression on offspring development has not been adequately addressed. One of the few studies conducted in this area (Field et al., 1993) suggested that the problematic interactions of depressed mothers with their infants are related to their negative perceptions of their infants' behaviour. These negative perceptions are consistent with the general cognitive biases that have been found to characterise depression (Gotlib and Joormann, 2010; Mathews and MacLeod, 2005). These biases operate most strongly when the cognitions involved are connected with interpersonal relationships (Gotlib and Hammen, 1992) and include distorted interpretation of interpersonal information, such as others' emotions and facial expressions (Bouhuys et al., 1995).

Given that the development of a strong mother–child relationship depends on a mother's ability to respond appropriately to her infants' cues – which are largely non-verbal – and that the processing of facial expressions is an essential feature in human interactions (Darwin, 1872/1955), it is likely that difficulties appraising infants' facial expressions underlie to a significant extent the problematic interactions between postnatally depressed mothers and their offspring.

A few studies have investigated the effects of depression on the processing of adult emotional facial expressions. Overall, results suggest that when compared to non-depressed participants, depressed individuals show deficits in processing emotional cues derived from facial expressions (Bouhuys et al., 1995; Cavanagh and Geisler, 2006; Coupland et al., 2004; Mogg et al., 2000a; Persad and Polivy, 1993). However, the majority of these studies have looked at full intensity (i.e., extreme) expressions and have generally reported mixed findings. For example, Leppänen et al. (2004) found that depressed individuals were as accurate as controls in identifying sad and happy faces, but were significantly less accurate in identifying neutral faces and attributed sadness to neutral facial expressions. Hale (1998) and Lee et al. (2008) also found that depressed individuals attributed significantly more sadness to ambiguous adult facial expressions than controls. However, Suslow et al. (2001) found that whereas depressed and control participants did not differ in their latencies to detect sad faces in a display of schematic faces, depressed participants were significantly slower than were controls to detect happy faces. Similarly, Gur et al. (1992) found that the tendency to misinterpret happy faces as neutral best discriminated depressed patients from controls.

A minority of studies have examined responses to lower-intensity expressions. As Joormann and Gotlib (2006) point out, processing of low-intensity emotional expressions and subtle changes in facial expressions of emotion may be stronger predictors of interpersonal functioning, given that in everyday life people are confronted with information comprising a wide range of emotional intensity, not only with full-intensity information. Results from research using low-intensity facial expressions have been fairly consistent in demonstrating that depression is associated with deficits in the processing of positive facial expressions (Deveney and Deldin, 2004; Yoon et al., 2009). These findings are in line with recent studies that suggest that depression is characterised primarily by difficulties in the processing of positive affect, perhaps even more than by biases in the processing of negative affect (e.g., Deveney and Deldin, 2004; Gilboa-Schechtman et al., 2002; Surguladze et al., 2004). Surguladze et al. (2004), for example, found that at 2-second presentations, depressed participants were less likely than their nondepressed counterparts to label 50% of happy faces as happy.

One task that has been used recently to examine the effects of mood on processing of facial expressions, including low-intensity expressions, is the ‘morphed faces task.’ This task, developed by Niedenthal et al. (2000), represents an alternative to the typically used prototypical or static faces. In everyday life, expressions are constantly changing and the ability to decode dynamic facial signals is crucial for social adaptation. Therefore, the morphed faces task mimics real-life processes by presenting a sequence of faces that change gradually between two emotions, typically between a neutral and a sad, happy, or angry expression (Heuer et al., 2010).

Findings from research using the morphed faces task have documented the impact of depression on the processing of happy faces. For example, studying healthy university students, Coupland et al. (2004) found that mood was associated with emotion identification. Specifically, whereas level of positive affect predicted participants' threshold for the identification of happy morphed faces, level of negative affect was associated with the threshold for the identification of disgusted morphed faces. Similarly, Cavanagh and Geisler (2006) showed that, overall, university students were more accurate in identifying happy than sad morphed faces and that those with higher levels of depression had greater difficulty identifying happy faces. In particular, they were slower to process this emotion, especially if the expressions were shown at lower intensities (e.g. 40%). Finally, Joormann and Gotlib (2006) found that whereas clinically depressed participants required significantly greater intensity of emotion than participants with social phobia and control participants to correctly identify happy facial expressions, participants with social phobia needed less intensity to correctly identify angry facial expressions than did depressed and control participants.

To date, virtually all of the studies examining cognitive biases in depression and the processing of facial expressions, including the morphed faces task, used adult faces as stimuli; consequently, little is known about biases in the processing of infant facial expressions. This is particularly important because infant faces have a different configuration than adult faces, including a relatively large head, predominance of the brain capsule, large and low-lying eyes and a bulging cheek region, which is posited to be important for eliciting parental responses (Lorenz, 1971). Two studies have examined responses to infant facial expressions in the context of depression. Pearson et al. (2010) found that whereas non-depressed pregnant women showed an engagement bias towards distressed infant faces, depressed women tended to disengage more quickly from the images. In addition, we found that mothers with postnatal depression were more likely to rate negative infant faces more negatively than controls, whereas mothers with postnatal GAD did not differ from controls (Stein et al., 2010). However, this study used faces that were at the extreme of the spectrum (i.e., obviously happy or sad), unlike the morphed face paradigm which utilises expressions at intensities more akin to ‘real life’, and did not attempt to explicitly measure accuracy. Given the crucial role of maternal responsiveness in later child development and the importance of processing infants' expressions in this dyadic interaction, we used a morphed faces task to examine the effects of maternal postnatal depression on the identification of emotional expression in infants' faces. In order to examine whether any obtained biases are specific to depression and not characteristic of more general postnatal psychiatric disturbance, we included a group of postnatal mothers who were experiencing anxiety. Although cognitive biases have been found in anxiety disorders, most typically those are specific attentional biases towards threatening stimuli rather than towards sad or more generally negative material (Mogg et al., 2000b).

We tested three hypotheses in this study. First, we predicted that all participants would classify happy faces more easily (i.e., with fewer errors and at a lower intensity) than they would sad faces. Second, based on findings from morphing studies using adult faces (Joormann and Gotlib, 2006; Surguladze et al., 2004), we predicted that depressed participants would exhibit a bias in the identification of happy faces, but not of sad faces. Finally, we hypothesised that this bias would be disorder-specific, such that compared to controls, anxious participants with Generalised Anxiety Disorder (GAD) would not exhibit differences in accuracy or intensity of identification of either sad or happy faces.

## 2 Method

### 2.1 Participants

Participants were part of the Oxford Parent Project (OPP). Mothers were recruited from the postnatal wards of the John Radcliffe Hospital, Oxford, UK. Inclusion criteria were i) 18 years or over; ii) sufficient level of English; iii) living within a 35 mile radius of Oxford; iv) no medical complications; v) principal infants' caretaker; vi) over 35 weeks gestation; vii) infant with birth weight of 2000 g and above, and viii) no life-threatening complication. All mothers who agreed to take part in the study were sent screening questionnaires (the Edinburgh Postnatal Depression Scale — EPDS, Cox et al., 1987; and the Generalised Anxiety Disorder Questionnaire — GAD-Q, Newman et al., 2002) at approximately nine weeks after giving birth. Those who scored above the threshold on either the EPDS (> 12) or GAD-Q (> 5.70), and a random sample of women who scored below the cut-off on both questionnaires were interviewed at home at 3 months using the Structured Clinical Interview for DSM (SCID; First et al., 2002, research version). The interviewer also rated the severity of the disorder using the Clinician's Severity Rating (CSR; DiNardo et al., 1993). Mothers who fulfilled the clinical criteria for Major Depressive Disorder (MDD) or Generalised Anxiety Disorder (GAD) in the diagnostic interview were considered eligible for the study. Inclusion criteria for control mothers included scoring below threshold in the screening questionnaires and no present or past psychiatric disorder. All mothers fulfilling the criteria for Major Depressive Disorder (MDD), Generalised Anxiety Disorder (GAD) and the controls (CON) were reassessed at 6 and 10 months postnatally. In addition, mothers with sub-threshold disorders (usually because the length of symptomatology did not fulfil the DSM criteria) or who were found to have had a history of depression or anxiety at the 3 month interview, were re-assessed at 6 and 10 months. If they met the DSM criteria at 6 or 10 months, they were included in the respective study groups. (One control participant who was originally included as a control at 3 months, was found to meet the diagnostic criteria for MDD at 10 months and removed from the control group and placed in the MDD group.) In the case of co-morbidity, group assignment was based on the principal diagnosis.

During the 18-month lab visit, the mothers in the MDD and GAD groups were reassessed using the SCID and completed the morphed faces task. Those mothers whose disorder was found to have remitted (CSR < 4) were not included in the morphed faces study. Due to the study design, control (CON) mothers did not attend the 18-month assessment; therefore the morphed faces task was completed in a prior visit, at about 10 months. The current study involved 89 mothers (21 MDD, 34 GAD, and 34 CON). The mean age of participants at the time of the assessment was 33.24 years (SD = 5.20) and the majority of mothers had completed a post 18-year old educational qualification. There were no differences between the groups on demographic variables (see Table 1 for sample demographic characteristics). Approval for the study was obtained from the Oxfordshire Research Ethics Committee. Informed consent was obtained from all participants.

### 2.2 Screening questionnaires

1. Edinburgh Postnatal Depression Scale (EPDS): the EPDS is a 10-item questionnaire for screening postnatal depression. Previous research has shown a sensitivity of 86% and a specificity of 78% as well as an acceptable internal consistency (Cronbach's alpha, α = 0.87; Cox et al., 1987).
2. Generalised Anxiety Disorder Questionnaire (GAD-Q): the GAD-Q is a self-report diagnostic questionnaire assessing GAD, as defined by the DSM-IV. The specificity and sensitivity have been reported to be over 80% and the internal consistency has been demonstrated to be good (α = 0.84) (Newman et al., 2002).

### 2.3 Interview

Structured Clinical Interview for DSM diagnosis (SCID): the SCID is a semi-structured diagnostic interview for assessing the DSM (First et al., 2002) Axis I disorders. At the end of the SCID interview a rating of the severity of the disorder was made using the Clinician's Severity Rating (CSR) (DiNardo et al., 1993). The CSR is a 0–8 scale indicating the level of distress and/or impairment associated with a given symptom cluster. A score of four or higher indicates clinical severity. Interrater reliability has been demonstrated for CSRs with kappa coefficients ranging from k = 0.67 to k = 0.86 for the various anxiety disorders (Brown et al., 2001).

### 2.4 Stimuli and procedure

The assessment took part in the research unit. The task was presented on a 12 in. laptop monitor placed approximately 38 cm from participants' eyes. Infant faces were drawn from a database of digital photographs of 27 infants who were filmed at home (Kringelbach et al., 2008) and were shown as greyscale images, matched for size and luminosity. A total of ten different faces, five sad and five happy, were presented in random order using ePrime software. Each face was morphed from neutral to 100% at increments of 2%. Altogether, 70 faces were presented even though only 50 differed in intensity: sometimes a face of a certain intensity was repeated multiple times before the screen moved on to the next intensity level to avoid having a perfect correlation between time and expression intensity and to increase the difficulty of the task (see Joormann and Gotlib, 2006, for details). Faces were shown for 500 ms.

Participants were required to press the space bar as soon as they thought they could identify the emotion that was being expressed. This stopped the sequence and they were then asked to label the emotion they had identified by pressing key 1 or 2 depending on whether they thought the face was happy or sad. These options were displayed in writing on the screen that appeared after the participants had pressed the space bar. There were two practice trials before the experiment.

## 3 Results

Responses given after 100% of the sequence had been completed were excluded from the analyses (39 responses, 1.1% of all trials). There were no significant group differences in the number of excluded trials, F(2, 86) = 0.56, *p* = 0.57.

We examined the hypothesis that all participants would classify happy faces more easily and at a lower intensity than sad faces (h1) using two-way Expression (happy vs sad) × Group (MDD, GAD, CON) repeated-measures analyses of variance (ANOVAs). Consistent with the suggestion of Rosenthal and Rosnow (1985) that the most appropriate way to test a priori predictions is by planned contrasts, these were conducted to test hypotheses 2 and 3. The first set of contrasts examined differences between depressed and control participants (contrast 1) and between anxious and control participants (contrast 2) in accuracy of happy and sad faces. The second set of contrasts involved corresponding contrasts for the intensity of happy and sad faces. One-tailed probabilities were used, because of these specific hypotheses which were based on the adult morphed faces literature, and alpha was controlled at *p* = 0.01 using a Bonferroni adjustment for each family of contrasts. To examine the potential confounding effect of accuracy on intensity of emotion identification, analyses for intensity were conducted both on all trials and on correct trials only.

### 3.1 Accuracy of identified faces

The repeated-measures ANOVA conducted on the accuracy scores yielded a main effect of Expression: participants had significantly lower error rates in identifying happy faces than sad faces, F(1, 86) = 46.14, *p* < 0.0001, η^2^ = 0.35 (happy average accuracy 91% (SD = 13); sad average accuracy 81% (SD = 11)) and this was consistent across groups (Group × Expression interaction: F(2, 86) = 1.54, *p* > 0.05, η^2^ = 0.03).

**Fig. 1:**
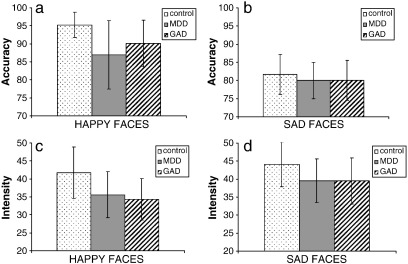
a) The percentage of accurately identified happy faces, b) the percentage of accurately identified sad faces, c) the intensity of identification of happy faces, and d) the intensity of identification of sad faces across control, MDD (Major Depressive Disorder), and GAD (Generalised Anxiety Disorder) groups. Note: error bars represent 95% confidence intervals.

Planned contrasts revealed that participants with depression were significantly less accurate in identifying happy faces than controls, t(86) = − 2.39, *p* = 0.005, η^2^ = 0.25, but participants with GAD were not significantly different from controls although they showed a trend in the same direction, t(86) = − 1.68, *p* = 0.05, η^2^ = 0.18 (Fig. 1a). With regard to sad faces, there were no significant differences between control participants and participants with GAD, t(86) = − 0.59, *p* > 0.01, η^2^ = 0.06, or between control participants and participants with GAD, t(86) = − 0.67, *p* > 0.01, η^2^ = 0.07 (Fig. 1b).

### 3.2 Intensity of emotion identification

The repeated-measures ANOVA conducted on the intensity of expression required to identify the facial emotions yielded a significant main effect of Expression, F(1, 86) = 30.75, *p* < 0.0001, η^2^ = 0.26. Participants identified happy faces earlier in the sequence (i.e., at a lower intensity) than they did sad faces (happy M = 37.48 (SD = 13.27); sad M = 41.25 (SD = 12.57)) and this was consistent across groups (interaction of Group and Expression: F(2, 86) = 1.78, *p* > 0.05, η^2^ = 0.04).

Somewhat contrary to expectations, participants with GAD happy faces at a significantly lower intensity than controls, t(86) = − 2.37, *p* = 0.005, η^2^ = 0.25 (Fig. 1c). Whilst not significant, there was also a trend for control participants and participants with depression to differ in the intensity at which happy faces were identified, t(86) = − 1.73, *p* = 0.04, η^2^ = 0.18, with participants with depression identifying happy faces at a somewhat lower intensity than controls. As far as sad faces were concerned, there were no significant differences between control participants and participants with depression, t(86) = − 1.31, *p* > 0.01, η^2^ = 0.14, or between control participants and participants with GAD, t(86) = − 1.51, *p* > 0.01, η^2^ = 0.16, in the intensity at which they were identified (Fig. 1d).

Analyses on correct trials only, yielded a similar pattern of results for intensity of identification of happy faces; participants with GAD identified happy faces at a significantly lower intensity than control participants, t(86) = − 2.31, *p* = 0.005, η^2^ = 0.24, and participants with depression, compared to control participants, showed a nonsignificant trend in the same direction, t(86) = − 1.71, *p* = 0.05, η^2^ = 0.18. Moreover, similar to the findings on all trials, analyses of sad faces revealed no significant differences between controls and either participants with depression, t(86) = − 1.20, *p* > 0.01, η^2^ = 0.13, or participants with GAD, t(86) = 1.54, *p* > 0.01, η^2^ = 0.16.

## 4 Discussion

This study was designed to use a morphed faces task to investigate the effects of maternal postnatal depression on recognition of emotion in infant faces. Consistent with previous work using adult faces (Cavanagh and Geisler, 2006), participants overall were more accurate in identifying happy infant faces than they were in identifying sad infant faces. In addition, we did not find evidence that having postnatal depression or anxiety had effects on accuracy of identification of sad faces. In contrast, mothers with depression were less accurate than controls in identifying happy faces. These findings are consistent with studies examining responses to full-intensity facial expressions (Gur et al., 1992; Suslow et al., 2001) and most importantly with research on responses to low-intensity and subtle adult emotion expressions (Cavanagh and Geisler, 2006; Joormann and Gotlib, 2006; Surguladze et al., 2004). This is particularly important because if mothers with depression have difficulty identifying their infants' happy faces, this may make their responses to their infants less positive and more negative (Field et al., 1993). This finding raises a possible mechanism by which depression leads to negative maternal responsiveness (Murray et al., 1993; Stanley et al., 2004) and, thereby, to later difficulties in child development (Yarrow et al., 1984). We also found that mothers with GAD showed a trend in the same direction as mothers with depression in that they were somewhat less accurate than controls in identifying happy faces. This suggests that the difficulties in identifying happy faces accurately may not be specific to depression.

Our findings that mothers with depression did not respond differently to sad faces compared to controls are also in keeping with research on responses to low-intensity and subtle adult emotion expressions (Cavanagh and Geisler, 2006; Joormann and Gotlib, 2006; Surguladze et al., 2004). It may be that mothers with depression only respond differently from controls when presented with extreme negative infant emotions for longer periods of time (Stein et al., 2010).

Interestingly, mothers with GAD were found to identify happy faces at a lower intensity than controls. Conceivably, such enhanced sensitivity to happy facial expressions may be a function of a search for reassurance from their environment that negative events will not occur, given evidence for their characteristic intolerance of uncertainty (Ladouceur et al., 1999) and seeking of reassurance due to their worries (Townsend et al., 1999). Indeed, repeatedly seeking reassurance due to worries is currently proposed as a key diagnostic feature for GAD in DSM V (Andrews et al., 2010). The urgent need of individuals with GAD to be reassured that negative events will not occur may therefore lead to an increased sensitivity to happy or positive signals — in this case, happy emotion expressions. Thus, a mother with GAD may be particularly prone to identifying happy infant faces as such facial expressions lessen her anxiety and worries about parenting behaviours. There was also a trend for mothers with depression to identify happy faces at a lower intensity than controls. More research, including replication of this finding, is required in order to interpret this result.

There were a number of limitations of this study. First, the number of mothers in each group was not large and the findings will need replication. Second, as a result of the small numbers, it was not feasible to conduct more complex analyses, in particular to explore group (MDD, GAD or control) by emotional face (happy/sad) interactions, to examine these data in more detail. Third, because of our study design, control mothers completed the task when their children were several months younger than the children of the index mothers. However, because mothers become more familiar with infants' emotions with age, the differences between controls and depressed and GAD mothers would likely to have been stronger had the control mothers performed the task at the same time as mothers with depression and GAD. Fourth, studies with larger numbers may also be required to assess the effects of co-morbidity.

In conclusion, we found in this study that mothers of young children were generally more sensitive to happy than to sad infant faces. However, mothers with postnatal depression were less likely to correctly identify happy faces than were control participants. Interestingly, mothers with GAD identified happy faces at a lower intensity than controls, which is consistent with the literature demonstrating that individuals with GAD are intolerant of uncertainty and seek reassurance due to their worries. To our knowledge, this is the first study to utilise infant morphed faces to illuminate early parental responsiveness and, in particular, to examine the effects of postnatal depression on the processing of infant facial expressions. These results have the potential to address important questions about how a negative cognitive bias may affect depressed mothers' perceptions of their infants' emotions. This is the first study of its kind, and further research using larger samples is required to explore these issues more systematically. If the present findings are replicated, research is needed to investigate the links between mothers' perceptions of infant facial expressions and both maternal responsiveness and later measures of child development.

## Role of funding source

The funder had no role in study design, data collection, data analyses, data interpretation or writing of the report.

## Conflict of interest

The authors do not have any conflict of interest.

## Figures and Tables

**Table 1:** Demographic characteristics of the groups.

		Control (*n* = 34)	MDD (*n* = 21)	GAD (*n* = 34)
Maternal qualification (%)	No post 18 year qualification	23.5	40.0	35.3
	Post 18 year qualification	76.5	60.0	66.7
Mother age yrs (mean/SD)		33.56 (4.32)	32.33 (6.54)	33.50 (5.20)
Infant sex (% boys)		38.2	38.1	52.9
Parity (% first born)		66.7	60.0	57.7
Baby birth weight kg (mean/SD)		3.37 (0.63)	3.38 (0.51)	3.42 (0.56)

MDD = Major Depressive Disorder. GAD = Generalised Anxiety Disorder.
